# Transient responses of double core-holes generation in all-attosecond pump-probe spectroscopy

**DOI:** 10.1038/s41598-024-52197-y

**Published:** 2024-01-23

**Authors:** Jianpeng Liu, Yongqiang Li, Yong Hou, Jianhua Wu, Jianmin Yuan

**Affiliations:** 1 College of Science, National University of Defense Technology, Changsha, 410073 China; 2 Hunan Key Laboratory of Extreme Matter and Applications, National University of Defense Technology, Changsha, 410073 China; 3 Department of Physics, Graduate School of China Academy of Engineering Physics, Beijing, 100193 China; 4 Institute of Atomic and Molecular Physics, Jilin University, Changchun, 130012 China

**Keywords:** Atomic and molecular interactions with photons, Attosecond science, Exotic atoms and molecules

## Abstract

Double core-holes (DCHs) show remarkable and sensitive effects for understanding electron correlations and coherence. With advanced modulation of x-ray free-electron laser (XFEL) facility, we propose the forthcoming all-attosecond XFEL pump-probe spectroscopy can decipher the hidden photon-initiated dynamics of DCHs. The benchmark case of neon is investigated, and norm-nonconserving Monte-Carlo wavefunction method simulates non-Hermitian dynamics among vast states, which shows superiority in efficiency and reliability. In our scheme, population transfer to DCHs is sequentially irradiated by pump and probe laser. By varying time delay, Stark shifts and quantum path interference of resonant lines sensitively emerge at specific interval of two pulses. These ubiquitous multi-channel effects are also observed in phase-fluctuating pulses, derived from extra phases of impulsive Raman processes by pump laser. Non-perturbation absorption/emission verifies the uniquely interchangeable role of two pules in higher intensity. Our results reveal sensitive and robust responses on pulse parameters, which show potential capacity for XFEL attosecond pulse diagnosis and further attosecond-timescale chemical analysis.

## Introduction

The double core-holes (DCHs) represent exotic electronic states of double core vacancies in atoms or molecules, first elaborated by Cederbaum et al. ^1^ in early 30 years. Due to that double ionization potentials are sensitive to valence-electron correlations in molecules, these hollow species draw much attentions on the formation, energy and subsequent Auger decay at synchrotron radiation experiments ^2–7^. Recently, the inspiring x-ray free electron laser (XFEL) facilities generate pulses with unprecedentedly temporal and spatial resolution. Sequential ionization dominates dynamics of DCHs generation via single core-holes (SCHs) by two-photon absorption. Once induced photoionization surpasses Auger decay, production of DCHs in time domain can be observed ^8,9^. It has been proposed as a novel tool of chemical analysis based on electron spectroscopy ^10–17^ and investigation on non-linear x-ray interactions ^18–21^. On account of both two extremely unstable core-holes, detailed analysis on transients of these intermediate and final states has been implemented in static domain ^20,21^. Recently, femtosecond (fs) many-body correlations of molecules’s SCHs have been investigated in a site-selective pump-probe scheme ^22^, as well as electron coherence in impulsive stimulated Raman scattering ^23^. These complementary advances shed light on the fundamental photoinduced transients of inner-shell electrons in other subjects ^24–27^.

Referring to time-resolved technique, attosecond (as) lightsources and “as pump + fs probe” measurements show excellent opportunities for investigating fs/as dynamics in table-top facility ^27–29^, while the desiring all-attosecond scheme is limited by flexible modulation of two controllable as pulses. Recently, as x-ray lasers with high brightness, short wavelength and short duration have been realized in XFELs facility ^30–33^, and two-color pulses with controlled wavelength, intensity and timing have been produced ^33–35^. Other elaborate modulations are introduced for new x-ray as lightsource ^36–39^. Transient absorption by all-extreme-ultraviolet pump-probe technique has also been implemented for unveiling valence-shell electron dynamics in fs timescale ^40–42^. All of these impressed improvements take a solid step for *all-attosecond XFEL pump-probe spectroscopy* (AXPPS) ^33^, which becomes an upgrade XFEL project for future user experiments ^43,44^.

Despite currently remaining challenges in AXPPS, this spectroscopy manifests a desirable tool for studying dynamics of unstable states within as timescale. In this work, we benchmark fundamental transients of DCHs generation induced by two controllable x-ray lasers, and take DCHs of Ne’s 1*s*-shell as an example. Despite of closed-shell structure, our results show relevant multi-channel effects and diversely subsequent decay processes are indispensable in transient responses, of which the few-level model is inadequate to describe the real dynamics: First pump pulse creates Ne$^+$ SCHs $1s^1$, and then probe pulse tunes to resonance transitions of Rydberg series DCHs $1s^1 \leftrightarrow 1s^0\text {n}p$ ^21^. By precisely varying time delay, resonant lines with shifts, broadening and beating retrieve the hidden attosecond-timescale dynamics, accompanied with impulsive Raman processes and relaxation of Auger decay, photoionization, spontaneous decay etc ^45^. Consequently, large-scale simulations based on quantum master equation (QME) are demanded in ultrafast dynamics of complex systems with strong dissipation ^46,47^. For computational efficiency, Monte-Carlo sampling on plenty of transition pathways is introduced and adapted in this work ^48–51^. Our results show reliable dependence on pulse parameters, indicating an exciting test case for XFEL double-pulse diagnosis, especially for timing modulations, which has not been utilized ^33^. The evident responses of DCHs generation also show potential capacity for next-generation attosecond-timescale chemical analysis.

This paper is organized as follows. Section of “Results and discussions” covers our results and discusses transient absorption/emission of DCHs generation in AXPPS. Section of “Summary and outlooks” summarizes as well as presents the potential applications of DCHs. In Section of “Methods” we present Monte-Carlo wavefunction (MCWF) method and its fidelity of applications to transient spectroscopy of DCHs. Supplementary information (SI) presents details of atomic data and proofs. Notice that atomic units are used throughout paper unless indicated otherwise.

## Results and discussions

**Figure 1:**
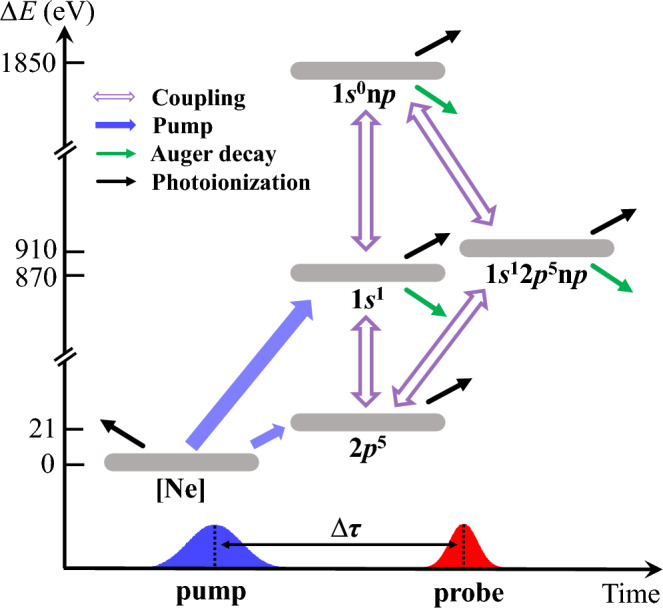
Sketch of generating Ne 1*s*-shell DCHs in AXPPS. For convenience, only relevant configurations of gray bars are listed, e.g. [Ne], $2p^5$, $1s^1$, $1s^0\text {n}p$ and $1s^12p^5\text {n}p$ with $\text {n}=3-6$. Blue fulled arrows show pump processes from Ne to Ne$^+$. Purple hollow arrows show coupling channels by both two pulses. Green and black solid lines show Auger decay and further photoionization, respectively.

The involved processes see in Fig. 1, transitions of Auger decay (green arrows) and further photoionization (black arrows) to other irrelevant states introduce population leak into dynamics. The norm-nonconserving formula is adapted by eliminating the first part in bracket of Eq. (4) ^52^ (see “Methods”).

### Population transfer and transient absorption spectrum

**Figure 2:**
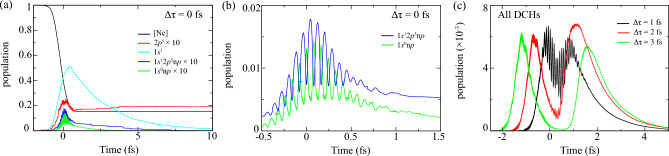
Time-dependent population *P*(*t*) at fixed pump-probe time delay $\Delta \tau$. (**a**) Shows all relevant 5 configurations at $\Delta \tau =0$ fs. And (**b**) enlarges the evolution curves of SCHs $1s^12p^5\text {n}p$ and DCHs $1s^0\text {n}p$ at $-0.5~\text {fs}<t<1.5~\text {fs}$. (**c**) Shows total yield of DCHs at $\Delta \tau =1,2,3$ fs. Notice that curves of $2p^5$, $1s^12p^5\text {n}p$ and $1s^0\text {n}p$ are magnified by 10 for clarity, and error bar of DCHs’ population in (**b**) is presented compared with QME’s results.

Firstly, we present time-dependent population for tracing dynamics irradiated by two pulses. Sequential picture in Fig. 2(a) of DCHs generation is deciphered as follows: Significant ionization occurs from ground state to SCHs $1s^1$ by pump laser. In the mean time, probe laser induces fast Rabi oscillation of transitions to DCHs $1s^0\text {n}p$ with period 100 as during the overlap of $-0.5$ fs $<t<1$ fs, resulting in $5\times 10^{-3}$ retained at the tail shown in Fig. 2b. Due to Raman channels embedded in DCHs generation, part of population transfer $1s^0\text {n}p \rightarrow 1s^12p^5\text {n}p$ occurs with inverse oscillating phase. Besides, as can see dynamics at larger $\Delta \tau$ in Fig. 2c, population of DCHs can also be transiently excited, adiabatically followed the Gaussian profile of pump laser and then quickly dropped. It derives from coherent and off-resonant dynamics in Raman channels, which may influence the abundance and lineshape of DCHs’ Auger spectrum ^21^. When evolution time exceeds duration of two pulses, dissipative processes dominate the evolution in the range out of pulse duration. Exponential decay of SCHs and DCHs occurs via Auger decay channels and the ground state Ne$^+$ $2p^5$ remains without further photoionization.

**Figure 3:**
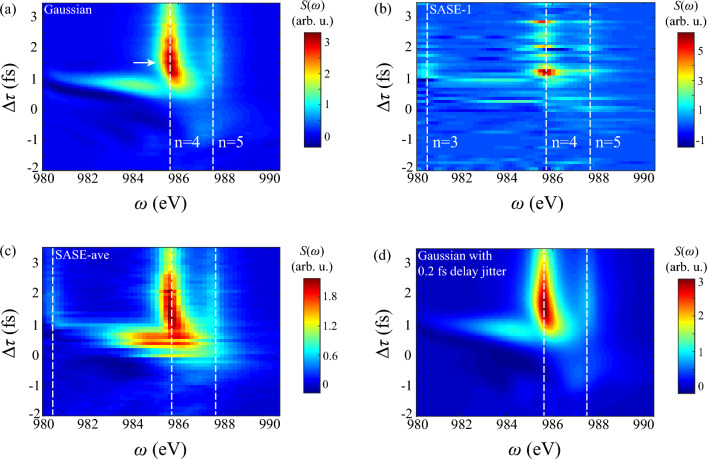
Transient absorption spectrum of DCHs generation in AXPPS. X- and y-axis show photon energy and $\Delta \tau$, respectively. $\Delta \tau >0$ labels pump laser arrives before probe laser. (**a**) Shows results of Gaussian-profile pump and probe lasers. (**b**) presents single-shot spectrum of SASE-mode pulses with spike $\tau _s=0.26$ fs at each $\Delta \tau$. (**c**) Shows 100-averaged results of SASE-mode pulses. And (**d**) shows 200-averaged results of Gaussian-profile pulses with 0.2 fs delay jitter. White dashed lines identify resonant transitions of DCHs and white arrow shows maximum absorption peak at $\Delta \tau =1.5$ fs in panel (**a**). Notice that value in all absorption spectrum of this paper is estimated by absolute strength.

Figure 3 illustrates attosecond-timescale absorption in AXPPS. Here one step of $\Delta \tau$ is 100 as. Positive delay represents pump laser arrives before probe laser. Conversely, negative delay represents probe laser arrives before pump laser. In Fig. 3a strong absorption around 986 eV emerges at positive delay, corresponding to resonant transitions $1s^1$ $^2$S$_{1/2}\leftrightarrow 1s^0 4p$ $^2$P$^\text {o}$. Natural width of resonant line is about 0.6 eV mainly determined by Auger decay and photoionization. In the range of $\Delta \tau >1$ fs, absorption effect weakens due to strong Auger decay damps coherent dynamics and residual population of SCHs. At $\Delta \tau =1.5$ fs, the absorption reaches to maximum (pointed by white arrow), indicating retarded time between population transfer to SCHs $1s^1$ $^2$S$_{1/2}$ and coherent absorption of DCHs. Similar effect is presented between establishment of population inversion and lasing processes in atomic inner-shell X-ray laser ^53^. Besides, nearly transverse absorption line occurs at around $\Delta \tau =1$ fs. And resonant line shows distinct S-type curve from negative to positive delay. Both two effects derive from impulsive Raman processes, and detailed analysis sees in next subsection.

Now we discuss the influence of fluctuating pulses on pump-probe scheme. The present-day XFELs at short wavelength are mostly based on self-amplified spontaneous emission (SASE) mode, where pulses have stochastic sub-pulses known as multi-spike structures ^54^. Therefore, instabilities of temporal profiles of each pulses and effective $\Delta \tau$ both emerge in pump-probe scheme, which may strongly cover up transients of DCHs. Figure 3b illustrates the SASE result without averaging at each $\Delta \tau$, corresponding to experimental observation. Due to random SASE pulses of pump and probe lasers at each $\Delta \tau$, fluctuating pattern of resonant lines emerges at $\Delta \tau >1$ fs, which shows fundamental features of transient absorption can be confirmed. While in Fig. 3c, one can see 100-averaged simulation in a trace resembles the Gaussian-profile case at $\Delta \tau >1$ fs, with similar trend of shifts and broadening in resonant lines. The distinct difference is the length of response region and strong absorption with randomness at around $\Delta \tau =0$ fs. Due to larger spectral width of SASE pulses $\Delta \omega _s=4 \text {ln}2/\tau _s \approx 7.2$ eV, signal of DCHs $1s^0 3p$ is evident in spectrum. On the other hand, fluctuated SASE-mode temporal profile contributes to evident absorption during the overlap, which also appears in Fig. 7b and Fig. 8b of higher intensity of probe and pump laser, respectively. It indicates the instability of intensity of sub-pulses cause this phenomenon.

As for the role of delay jitter of two pulses, Fig. 3d illustrates 200-averaged results of Gaussian-profile pulses with extra stochastic delay jitter. Here we introduce random interval from normal distribution with standard deviation 0.2 fs at each specific $\Delta \tau$ point, based on the current split-undulator technique ^33,55^. Notice that only randomness is $\Delta \tau$, while the evolution dynamics irradiated by ideal pulses is deterministic. Therefore, averaged results are the convolution of spectrum in Fig. 3a and Gaussian-window function of 0.2 fs width along delay axis. Simulation results in Fig. 3c show similar responses with vertical “S”-type resonant lines and slightly indistinct transverse absorption line from $1s^03p$ at positive delay, due to jitter is quite smaller than characteristic time in DCHs’ responses. The latter phenomenon also occurs in SASE-mode results, since randomness of sub-spikes also disturbs the effective interval of two pulses.

Above all, transient spectrum of DCHs illustrates abundant ultrafast responses in AXPPS, and fidelity of these effects has been proved even with fluctuating intensity and delay jitter.

**Figure 4:**
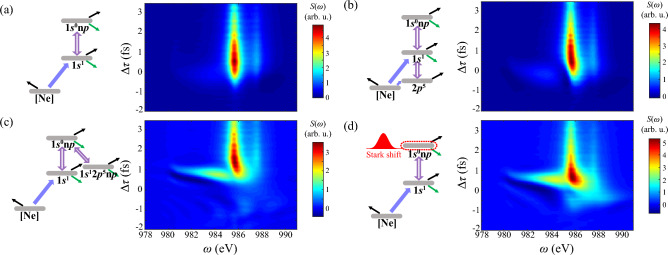
Transient absorption spectrum of DCHs generation in different models. Parameters of two pulses are consistent with Fig. 3. (**a**) Shows results only including contributions of SCHs $\leftrightarrow$ DCHs. (**b**) Shows results excluding $1s^12p^5\text {n}p$. (**c**) Shows results excluding $2p^5$. And (**d**) shows results by incorporating Stark shifts into sole SCHs $\leftrightarrow$ DCHs adiabatically.

### Role of impulsive Raman processes

Previous illustrations show evident energy shifts and distortion of resonant lines during the overlap. For detailed investigation on these responses, simulations eliminating relevant states of Raman processes are shown in Fig. 4.

We first consider sole DCHs transitions $1s^1$ $^2$S$_{1/2}\leftrightarrow 1s^0 \text {n}p$ $^2$P$^\text {o}$ without additional channels in Fig. 4a. It clearly shows two vertical resonant lines $\text {n}=4,5$ when $\Delta \tau >-1$ fs. This effect is reliable since no extra phase incorporates into resonant transition and correspondingly no energy level is perturbed. Next, non-resonant transitions $1s^1 \leftrightarrow 2p^5$ are incorporated and fulfills the cascading three-level model in Fig. 4b. Results show line bends to higher energy at $\Delta \tau = -0.5$ to 1 fs, indicating Stark shift derives from strong pump laser: At around zero delay, pump-induced Stark shifts in coherent ultrafast excitation to DCHs is incorporated, adiabatically followed Gaussian profile. Similar symmetric and slight shifts during the overlap can be found in all-extreme-ultraviolet XFEL pump-probe scheme ^40^. The reason of positive shifts can be qualitatively explained by cascading three-level model in SI. In addition, symmetric Autler-Townes splitting around $\Delta \tau =0$ fs is absent, since non-resonant coupling is introduced by pump laser. When $\Delta \tau >1$ fs, two pulses have no overlap and strong decay of SCHs and DCHs damps these phase modulations. This phenomenon is different with conventional valence-shell dynamics, where long lifetime of excited states retains coherence for larger $\Delta \tau$ ^56^.

Furthermore, Fig. 4c illustrates more complicated multi-channel $\Lambda$-type model including $1s^0 \text {n}p \leftrightarrow 1s^12p^5 \text {n}p$. Positive Stark shift of $1s^04p$ at $\Delta \tau =$ 0.5 fs also occurs, consistent with effects in Fig. 4c. It can be explained by $\Lambda$-type three-level model in SI. The distinction is the nearly transverse absorption line at around $\Delta \tau =1$ fs between $1s^03p$ and $1s^04p$, while much weaker line locates at $\Delta \tau =0$ fs in cascading three-level model. These long-range responses in non-resonant region are also observed in supercontinuum lasing driven by a strong laser field ^57^, due to multi-level synergistic effects. We can conclude this phenomenon derives from stronger Stark shift of DCHs $1s^03p$ via multi-channel $\Lambda$-type Raman processes, where oscillation strength of $1s^03p$ transition is 5 times larger than $1s^04p$. Since there is no significant variance between results of Figs. 4a and 3a, main Stark shifts in complete model attribute to $\Lambda$-type two-photon Raman processes $1s^1 \leftrightarrow 1s^0 \text {n}p \leftrightarrow 1s^12p^5 \text {n}p$. It is obtained that smaller detuning and plenty of transitions $1s^0 \text {n}p \leftrightarrow 1s^12p^5 \text {n}p$ dominate non-linear perturbation. One thing should be noticed that plenty of inter-coupling channels $1s^0 \text {n}p \leftrightarrow 1s^12p^5 \text {n}p$ would introduce higher-order Stark shifts and complicate explicit expressions. For clarification of this non-resonant coupling, we simplify coupling processes and deliberately incorporate time-dependent Stark shifts into DCHs transitions in Fig. 4d, of which transition energy has form

1 $$\begin{aligned} \Delta \tilde{E}(t)=\Delta E + \overline{\delta E}(t),~\overline{\delta E}(t) >0. \end{aligned}$$

Here $\Delta E$ and $\overline{\delta E}(t)$ represents field-free transition energy and averaged Stark shifts induced by pump laser, respectively. This adiabatic manipulation introduces extra dynamical phases into transitions. Results show analogical nonlinear effects compared with Fig. 3a, which emphasize Stark shifts from extra non-resonant coupling. Nevertheless, $\Delta \tau$ of maximum absorption retards in the real simulation. It indicates that non-adiabatic effects of population transfer to $1s^12p^5\text {n}p$ by pump laser postpones DCHs’ resonant absorption.

**Figure 5:**
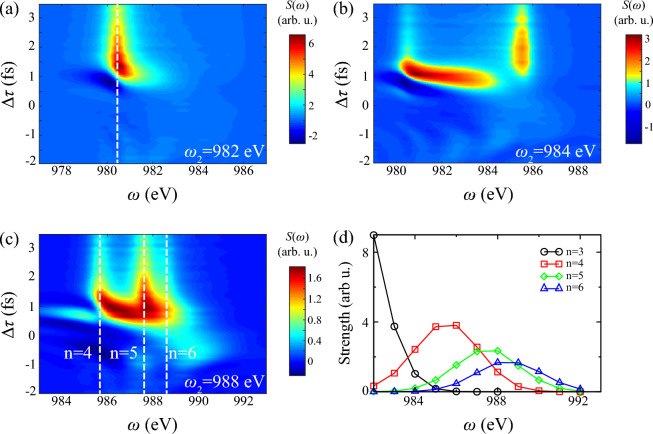
Transient absorption spectrum and integrated function of DCHs generation with different photon energy of probe laser $\omega _2$. Other parameters are consistent with Fig. 3. Here $\omega _2=$ (**a**) 982 eV, (**b**) 984 eV, (**c**) 988 eV. Panel (**d**) shows integrated absorption strength of four Rydberg series lines $1s^0\text {n}p~(\text {n}=3-6)$ as the function of $\omega _2$, with area $-2$ fs $<\Delta \tau<$ 3.5 fs and 0.6 eV natural width. White dashed lines identify resonant transitions of DCHs.

### Parameter dependence on probe laser

In the well-developed “as pump + fs probe” spectroscopy, electron dressed by infrared fs laser is transiently excited by as pulses ^56^, with significant difference of two pulses in temporal and spectral domain. While in AXPPS, pump injection by photoionization, electron excitation by probe laser, dissipative transition, etc occur in hundreds of as. The role of probe laser should be carefully investigated since two pulses have no significant difference in duration and wavelength.

Figure 5 illustrates dependence of $\omega _2$ on absorption spectrum and integrated function. Figure 5a–c show distinct spectrum with resolved Rydberg states $1s^0\text {n}p~(\text {n}=3-6)$. Positive shifts of all states emerge around zero $\Delta \tau$, which proves the conclusion of simplified Raman model: The sign of Stark shift only depends on the sign of detuning between pump laser and Raman transitions. Figure 5b shows strong Stark shift of $1s^0 3p$ with 4 eV extension to $1s^04p$, consistent with absorption signals from $1s^0 3p$ in non-resonant region in Fig. 3a. Furthermore, we summarize integrated signals along with $\Delta \tau$ as the function of $\omega _2$ with different DCHs in Fig. 5d. Here integrated width is the natural width of DCHs approximately 0.6 eV. It illustrates energy-resolved peaks of Rydberg series DCHs are presented when varying $\omega _2$. Notice that weaker absorption occurs in higher DCHs, and their bandwidth $\Delta \omega \approx 4$ eV is determined by convolution of spectral width of probe laser and natural width.

**Figure 6:**
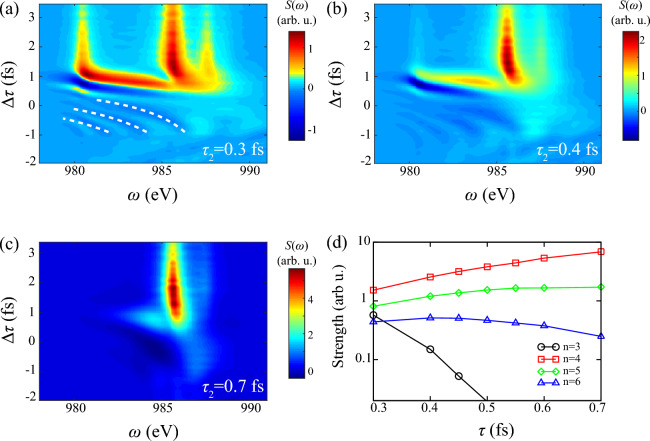
Transient absorption spectrum and integrated function of DCHs generation with different pulse duration of probe laser $\tau _2$. Other parameters of two pulses are consistent with Fig. 3. Here $\tau _2=$ (**a**) 0.3 fs, (**b**) 0.4 fs, (**c**) 0.7 fs. Panel (**d**) shows integrated absorption strength of four Rydberg series lines $1s^0\text {n}p~(\text {n}=3-6)$ as the function of $\tau _2$. The integrated area is same as Fig. 5. White dashed lines show points with equal phase.

Since duration of probe laser determines resonant range, we further present dependence of $\tau _2$ on absorption spectrum and integrated function in Fig. 6. One interesting phenomenon in Fig. 6a is that evident hyperbolic interference fringes occur at $-1$ fs $<\Delta \tau<$ 0 fs between $1s^03p$ and $1s^04p$, and gradually vanish in larger duration. We conclude it derives from quantum path interference of overlapped bound-bound transitions of diverse SCHs to the same shifted state $\{1s^1,1s^12p^5\text {n}p\} \rightarrow 1s^03p$. In negative delay during the overlap, population of $1s^1$ and $1s^12p^5\text {n}p$ is injected and transiently transferred by pump laser, then respectively excited to $1s^03p$ by probe and pump laser via different channels. Under this circumstance, accumulated phase difference attributes to different transition energy of SCHs. “Which-way” interference occurs and results in periodic oscillation of emission signals ($S(\omega )<0$) ^58–60^. While in positive delay, strong resonant absorption destructs the establishment of interference. This unexpected interference of bound-bound transitions is different from multiple-photon-assisted interference in the vicinity of threshold in conventional pump-probe scheme ^60^. To qualitatively describe the interference, beating signals with equal phases have the relation ^59^

2 $$\begin{aligned} (\omega -\omega _0)|\Delta \tau -t_0|+\Delta \phi =2\pi k. \end{aligned}$$

Here *k* is a positive integer. $\omega$ is shifted energy of transient $1s^03p$. And $\omega _0=\omega _2-\delta =990$ eV is subtraction of probe laser $\omega _2$ and averaged detuning of $1s^12p^5\text {n}p \rightarrow 1s^03p$. Hence $\omega -\omega _0$ represents total detuning of two type single-photon transitions. $t_0=1$ fs and $\Delta \phi =1.5\pi$ are respectively relevant to time of transition probability peaks and inherent phase difference, varied to fit simulations. Three white dashed lines $k=1,2,3$ show good consistence with dark area in Fig. 6a. The further quantitative estimation is a tough question on these multi-channel interference, of which lifetime is comparable with pulse duration.

When increasing $\tau _2$, Fig. 6a–c show that absorption area shrinks resonant region from $1s^0 3p-1s^0 6p$ to only $1s^0 4p$, and consequently weakens signals of Stark shifts and path interference. As for integrated absorption in Fig. 6d, positive work on electron rises with increment of $\tau _2$, while the shrink of resonant area significantly reduces energy transfer in DCHs $\text {n}=3$. For nearly resonant line $\text {n}=4$ with detuning $\Delta E=0.6$ eV, saturation effect presents in large $\tau _2$ due to limited population of SCHs.

**Figure 7:**
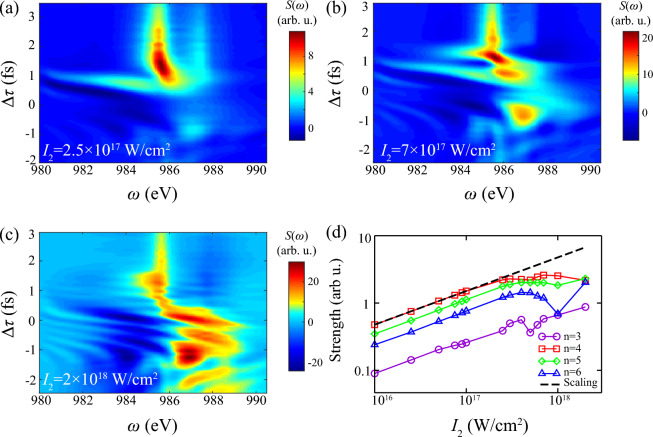
Transient absorption spectrum and integrated function of DCHs generation with different intensity of probe laser $I_2$. Other parameters of two pulses are consistent with Fig. 3. Here $I_2=$ (**a**) $2.5 \times 10^{17}$ W/cm$^2$, (**b**) $7 \times 10^{17}$ W/cm$^2$, (**c**) $2 \times 10^{18}$ W/cm$^2$. Panel (**d**) shows integrated absorption strength of four Rydberg series lines $1s^0\text {n}p~(\text {n}=3-6)$ as the function of $I_2$ in log-log diagram. The integrated area is same as Fig. 5. Notice that slope of black dashed line is 3/2 in log-log diagram.

In simulations as above, $I_2=0.1\times I_1$ is set to trace ultrafast responses under weak excitation, which has been widely implemented in conventional “as pump + fs probe” scheme. Since both two pulses have approximate wavelength and duration in AXPPS, probe laser can both induce photoionization to SCHs and two-photon Raman coupling when $I_2 \approx I_1$, Fig. 7 shows spectrum and integrated signal as the function of $I_2$. Compared with Figs. 3a and 7a, there is no significant variance since response is still in linear region. Furthermore, increment of $I_2$ shows distinct high-order effects, which probe laser contributes to Raman modulation and pump effects. Specifically, stronger Stark shift of $1s^03p$ emerges at $\Delta \tau =1$ fs and complex dipole responses during the overlap in Fig. 7b. And much larger shift and strong absorption/emission with Rabi oscillation in negative delay emerge when $I_2 > I_1$ in Fig. 7c. The integrated strength in Fig. 7d also indicates non-linear region emerges at $I_2 > 3\times 10^{17}$ W/cm$^2$, as complex absorption/emission signal shown in Fig. 7b, c. These unique signals show that role of pump and probe lasers is interchangeable in this setting, where stronger probe laser is capable to produce SCHs and absorption to DCHs, and succeeding pump laser induces nonlinear effects ^40^. The deviation of linear scaling law verifies complex non-linear phenomenon and saturation at higher intensity, which indicates the non-perturbation in AXPPS. And scaling law warrants transient features of transversely spatial-averaged results are dominated by parts at peak zone with higher signal strength.

### Parameter dependence on pump laser

**Figure 8:**
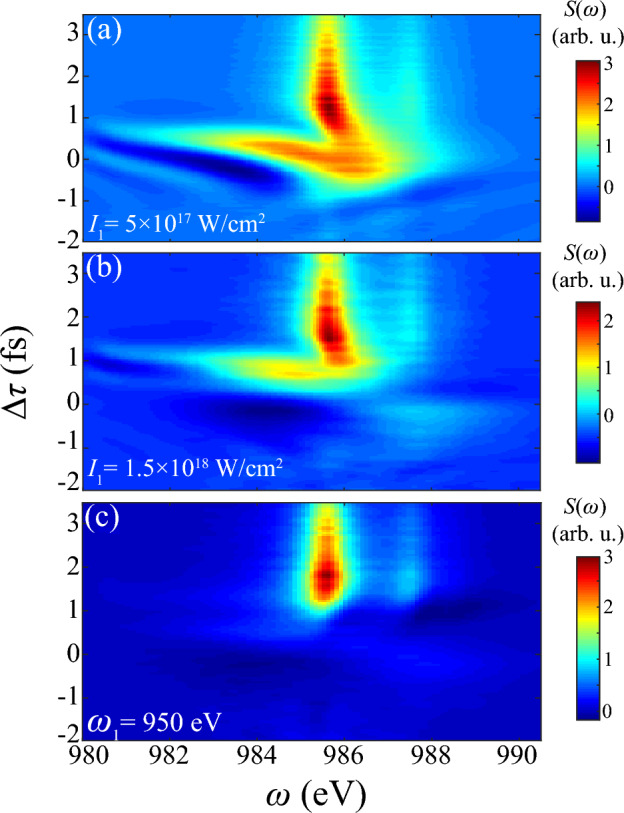
Transient absorption spectrum of DCHs generation with different parameters of pump laser. (**a**) $I_1=5\times 10^{17}$ W/cm$^2$, (**b**) $I_1=1.5\times 10^{18}$ W/cm$^2$ and (**c**) $\omega _1=950$ eV. Other parameters of two pulses are consistent with Fig. 3.

In DCHs generation of AXPPS, pump laser mainly provides population injection to SCHs and Stark shifts modulation, respectively determined by intensity and photon energy. For verification, dependence of these two parameters on transient spectrum is shown in Fig. 8. One can see both two cases of panel (a, b) show evident absorption within $|\Delta \tau |<1$ fs, derived from population transfer to shifted $1s^03p$ at different $\Delta \tau$. The transient energy of $1s^03p$ shifts to 986 eV at around zero delay when $I_1=5\times 10^{17}$ W/cm$^2$, following the significant population transition and absorption during the overlap. Similarly, in the case of $I_1=1.5\times 10^{17}$ W/cm$^2$, stronger shifted energy level matches to resonance at larger $\Delta \tau =0.7$ fs. While at $\Delta \tau >1$ fs, stark shift recedes and resonant absorption to $1s^04p$ still dominates. We can also remark these sensitive responses in simulations of SASE pulses, where the temporal fluctuation deviated from averaged value contributes to strong absorption during the overlap in Fig. 3c.

SI and Fig. 4c show the sign of Stark shifts mainly depends on detuning $\Delta =\omega _1-\Delta E$ of Raman channels $1s^0 \text {n}p \leftrightarrow 1s^12p^5 \text {n}p$. In above cases of $\omega _1=935$ eV, $\Delta \approx -8$ eV determines positive shifts during the overlap. Figure 8c with $\omega _1=950$ eV illustrates negative shifts of $1s^04p$ and $1s^05p$ in the condition of $\Delta \approx 7$ eV, consistent with our deduction. Besides, since sign of detuning inverses and transient energy of $1s^03p$ departs from resonant region, strong absorption of $1s^03p$ vanishes. These results enrich diverse pattern of transient responses and clearly show sensitivity and robustness on pump and probe lasers’ parameters.

### Emission properties of DCHs in AXPPS

**Figure 9:**
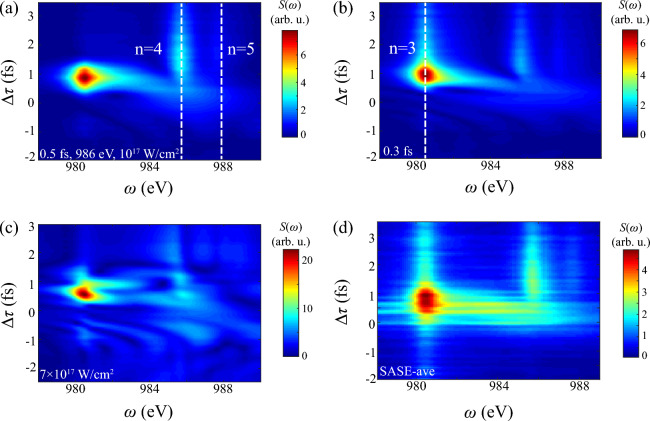
Resonant emission spectrum of DCHs with different laser parameters. (**a**) $\tau _2=0.5$ fs, $\omega _2=986$ eV and $I_2=10^{17}$ W/cm$^2$, consistent with Fig. 3a. (**b**) $\tau _2=0.3$ fs and (**c**) $I_2=7\times 10^{17}$ W/cm$^2$ show results of same parameters except labeled items. (**d**) shows averaged spectrum of SASE-mode with same input pulses parameters of panel (**a**). White dashed lines identify the position of respective radiation. Notice that value in this figure is estimated by absolute strength.

We turn to unravel emission properties of DCHs for completing transients in AXPPS. And also these sensitive phenomenon can capture tiny jitter of pulse parameters and benefit for XFEL as pulse diagnosis. Fig. 9 shows spectral dipole moment $|d(\omega ,\Delta \tau )|$ as the function of $\Delta \tau$. In contrast to absorption spectrum in Figs. 3a and 9a presents broader spectral profile even extending to $1s^03p$ around 980 eV. The evident emission signal of $1s^03p$ is observed in the vicinity of $\Delta \tau =1$ fs, indicating stronger population transfer. And this shift-induction transfer is blocked when overlap disappears at $\Delta \tau >1$ fs. It concludes the precise population transfer to specific DCHs is achieved by this delay-dependent manipulation. Stark shifts also contribute to broadband x-ray radiations between $1s^03p$ and $1s^05p$, which draws similar conclusion in ultraviolet air lasing ^57^. Emission signals of $1s^04p$ and $1s^05p$ at $\Delta \tau >1$ fs indicate characteristic radiation of DCHs’ dipolar oscillation after coherent excitation called free-induction decay (FID) ^57^, of which widths are mainly determined by Auger decay and photoionization. FID signal of $1s^03p$ is absent in Fig. 9a due to off-resonant condition, compared with emergence at $\tau =0.3$ fs in Fig. 9b. Figure 9c shows non-linear oscillating emission spectrum in higher intensity of probe laser. Besides, beating signals at negative delay also appear in emission spectrum, which excludes the possibility of optical interference ^60^ and verifies our conclusions of quantum path interference. Fluctuating effects in Fig. 9d show similar strong broadband emission signals during the overlap and FID signal at larger $\Delta \tau$ compared with absorption spectrum, respectively derived from high intensity of fluctuation and short duration of sub-spikes.

**Figure 10:**
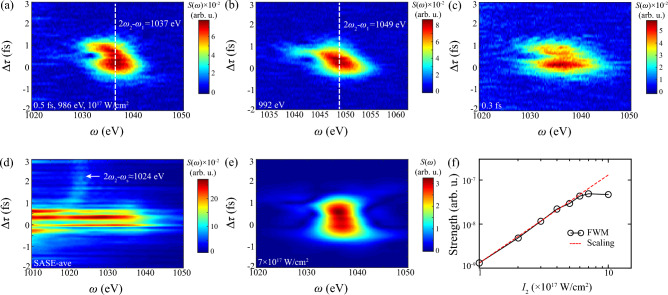
Delay-dependent four-wave mixing emission during DCHs’ generation with different probe laser parameters. (**a**) $\tau _2=0.5$ fs, $\omega _2=986$ eV and $I_2=10^{17}$ W/cm$^2$, consistent with Fig. 3a. (**b**) $\omega _2=992$ eV, (**c**) $\tau _2=0.3$ fs and (**e**) $I_2=7\times 10^{17}$ W/cm$^2$ show results of same parameters except labeled items. (**d**) Shows averaged spectrum of SASE-mode with same input pulses parameters of panel (**a**). And (**f**) shows integrated emission strength of four-wave mixing as the function of probe laser intensity with red-dashed line of slope 2. White dashed line and arrow identify the position of respective radiation. Notice that value in this figure is estimated by absolute strength.

One interesting phenomenon is that, under this strong dissipative quantum system, third-order x-ray nonlinearity of four-wave mixing (FWM) at $2\omega _2-\omega _1$ still emerges during the overlap in Fig. 10. In recent years, the well-established technique of dynamic (transient) gratings in X-ray region is based on periodic modulations of the sample’s optical properties by different excitations, which ultimately offers unique opportunities for probing novel processes in a wide variety of systems ^61–63^. In the fact of high flux of input pulses, AXPPS in this work can generate these high-order parts of polarization, beyond the limitation of phase-matching geometries of full-coherence fields. These robust and sensitive signals can capture prompt transients (phase, population, dipole moments, etc) of atoms or molecules in gas-phase medium and also make progresses on pulse diagnosis of developing as XFEL facility.

Figure 10a clearly illustrates radiation at $2\omega _2-\omega _1=1037$ eV, manifesting extra photon is emitted by absorbing two-photon of probe laser ($2\omega _2$) at $1s^1$ and then stimulated by pump laser ($\omega _1$) during the overlap. Besides, negative shift at large positive delay complies with trend of shifted energy level. It indicates that real transition $1s^1 \leftrightarrow 1s^0 3p$ occurs in the process of first photon absorption $\omega _2$, following Raman process $\omega _2-\omega _1$ and then emits FWM photon. We can also observe weak Stokes radiation $1s^0\text {n}p \rightarrow 1s^12p^5\text {n}p$ at $\omega _s \approx 940 \sim 950$ eV, which is not shown here. Results in Fig. 10b of $\omega _2=992$ eV verifies our deduction as the evidence of shifts of radiation position and distortion curve by diverse-DCHs-mediated FWM processes. And coherence feature of FWM allows resonant width $\sim 15$ eV in Fig. 10c (0.3 fs probe pulse) is larger than $\sim 9$ eV in Fig. 10a (0.5 fs). For comparison, we also present averaged radiation spectrum of SASE-mode in Fig. 10d and see much broad signals in the vicinity of zero similarly with Fig. 9d. The strong intensity fluctuation also contributes to slightly weak anti-Stokes radiation at $2\omega _2-\omega _s \approx 1024$ eV, due to that strong population transfer to final states $1s^12p^5\text {n}p$ via impulsive Raman channels ($\omega _2-\omega _s$) enhances anti-Stokes radiation. We verify our conclusion that signals at 1024 eV are absent in cascading three-level model (Fig. 4) with higher intensity. Finally, bending curve at $\Delta \tau \approx 1$ fs is absent at large intensity $I_2=7\times 10^{17}$ W/cm$^2$ of panel (e), indicating direct FMW channels dominate over indirect DCHs-mediated channels. The complex interference near the peak implies saturation of FWM, and integrated spectrum of log-log diagram in Fig. 10f completes responses of FWM processes.

### Feasibility of experimental observations

Now we show the feasibility of experimental observations on these transient responses. Ref. ^40^ presents sequential two-photon absorption of valence-shell electrons can be observed in XFEL pump-probe scheme, since its cross section is adequately large and sufficient ion abundance is obtained. According to Beer-Lambert law, generalized cross section of single atom’s absorption in SI units is given by ^64^

3 $$\begin{aligned} \sigma (\omega ,\Delta \tau )=\frac{\omega }{c\varepsilon _0}\frac{S(\omega ,\Delta \tau )}{|\mathscr {E}(\omega )|^2}, \end{aligned}$$

where *c* is the speed of light and $\varepsilon _0$ is dielectric constant in vacuum. The maximum value in Fig. 5a is about 0.5 Mb. Considering the experimental condition of 50 mbar pressure and 0.2 cm interaction length ^40^, the absorption ratio of probe laser is about 10%, within the spectral resolution in current experiments. Besides, emission spectrum apart from resonant region can be gathered by grating, for instance, strong signals of $1s^03p$ at 980 eV in Fig. 9a. This is well confirmed the point that DCHs’ transients can be observed in both absorption and emission spectrum.

As for part of pump-probe laser sources in current XFEL facility, Duris et al. ^33^ have reported split-undulator scheme in LCLS-II can generate pairs of synchronized pulses with tunable wavelength and duration, and also has the good performance in both spatial and temporal overlap at high intensities. This state-of-the-art technique can highly promote progress of x-ray “as pump + as probe” experiments. In our simulations, desirable transient effects are evident at above $I\sim 10^{17}$ W/cm$^2$. The corresponding focal diameter is about 10 µm based on available parameters about a peak power exceeding 100 GW and pulse duration down to 500 as of LCLS-II ^33^, which is within the current experimental feasibility.

## Summary and outlook

For unraveling hidden transient responses of inner-shell electrons irradiated by bright X-ray lasers, AXPPS on Ne’s 1*s*-shell DCHs is proposed and shows capacity of precise temporal and spectral resolution, with the advent of as pulses and two-color controllable mode in XFEL facility. The involved coherent Rabi dynamics with plenty of pathways between different fine-structure levels complicate the simulation, accompanied with strong incoherent cascade processes, e.g. photoionization, spontaneous decay and Auger decay. In this paper, we perform the large-scale norm-nonconserving MCWF for completeness properties of vast but not redundant states. And averaged results ensure both fidelity and time-consuming. Simulations clearly show delay-dependent spectrum with resolved Rydberg states even in SASE mode. Compared with simplified model and results of adiabatic approximation, we conclude Stark shifts derive from the coupling of extra Raman transitions. The interesting results are remarked that shift-assisted path interference can be established in bound-bound region under strong dephasing processes. Detailed investigations on influence of laser parameters show sensitive transient absorption in AXPPS, and diverse non-linear emissions present the extension of resonant dynamics to off-resonant region.

In this work, we observe significant absorption signals of DCHs with Stark shifts larger than 5 eV, which sensitively depend on parameters of induced x-ray pulses, e.g. energy, duration, intensity, delay, etc. This credible relation can be directly utilized to pulse diagnosis of forthcoming two controllable as x-ray laser in wide parameter region, by choosing appropriate atom’s or molecule’s DCHs. On the other side, electron spectrum of photoelectron or Auger electron of molecule’s two-site DCHs has become a reliable solution for chemical analysis, determining interatomic relaxation energy in static domain ^10^. Reference^22,65^ further demonstrates the pump-probe scheme for tracking time-dependent chemical shifts on the fs timescale, however, involving charge redistribution and nuclear motion indistinguishably. The disentanglement of electron and nucleus motion restricts temporal resolution down to as timescales ^23^. It has been shown that impulsive Raman processes can provide time-resolved nonadiabatic molecular processes in pump-probe x-ray absorption spectroscopy ^66^. In our simulations, since Stark shifts via pump-induced Raman processes introduce sub-fs and large-region absorption, e.g. distinct bending absorption curve in Fig. 5b, our scheme shows the potential applications on next-generation *attosecond-timescale chemical analysis*. The extension to other complex molecules is implemented by pumping one-atom SCHs and then probing other atoms’ core-valence transitions with impulsive Raman channels. During the overlap, nucleus motion is retarded and changes in energy and phase by extra valence-electron migration can be collected by high-efficiency transient absorption spectrum. Experimental value of this relaxation energy is about few eV ^16^, within the scope of Raman induced non-linear responses.

Besides, norm-nonconserving MCWF method will show incredible efficiency than QME in large-scale calculation of molecules, involving two atoms’s core-holes, diverse molecule’s valence-shell states and complex relaxation channels, which can extend dimension of configurations to ten thousands. The more robust MCWF method should be performed to present ab initio simulations of this non-Hermitian dynamics. Relevant work will be intended in the following paper. Our proposal allows us to unveil the hidden temporal multi-channel coherence, correlation-driven electron phenomenon and other interesting chemical dynamics via chemical shifts in as, and fulfills ongoing developments of new spectroscopy in XFEL facility.

## Methods

### Quantum master equation

The open quantum system theory deals with non-unitary evolution of quantum system coupling to reservoir, where an infinite number of variables complicates explicit calculation. QME traces out redundant degrees of freedom, transforming the system-reservoir interactions into dissipation or decoherence factors ^67–69^. For instance, the relevant resonant bound-free interactions, e.g. X-ray induced single-photon ionization, Auger decay, spontaneous decay of core-holes etc, are regarded as decoherent processes known as the famous Fermi’s Golden Rule ^67–69^. Under Born-Markov approximation, the evolution of reduced density matrix is governed by Lindblad-type QME ^70,71^

4 $$\begin{aligned} \frac{\partial }{\partial t} \hat{\rho }=- i [\hat{H}_s, \hat{\rho }]+\sum \limits _i {\frac{\Gamma _i}{2}\left( 2{\hat{\sigma }_i}\hat{\rho }\hat{\sigma }_i^\dag - \hat{\sigma }_i^\dag {\hat{\sigma }_i}\hat{\rho }- \hat{\rho }\hat{\sigma }_i^\dag {\hat{\sigma }_i}\right) }. \end{aligned}$$

Here $\hat{\rho }$ represents the time-dependent reduced density matrix of system. $\hat{H}_s$ is the total system Hamiltonian. $\Gamma _i$ represents the corresponding decay rate associated with channel *i*. And $\sigma _i$ denotes Lindblad operator. The first part of RHS in Eq. (4) represents the coherent evolution induced by x-ray lasers, and the second part shows Lindblad-type trace-preserving dissipation processes.

### MCWF sampling procedure and its deviation

Notice that it is a computationally intractable problem that variable number $N^2$ in density matrix obeys quadratic growth with number of basis set *N*. MCWF demonstrates high-efficiency performance based on important sampling in *N*-basis set ^72–74^. Besides, single trajectory in MCWF unravels the real-time evolution in dissipative surroundings, which brings new physical insights for non-Hermitian systems ^75–77^. Now we briefly present key ingredients of the Markovian MCWF method in norm-nonconserving condition.

Given normalized wavefunction $|\psi (t) \rangle$ at *t*, non-Hermitian Hamiltonian determines the effective evolution from *t* to $t+\delta t$, which reads

5 $$\begin{aligned} \hat{H}_e = \hat{H}_s - \frac{i}{2}\sum _m \hat{C}_m^\dagger \hat{C}_m . \end{aligned}$$

Here $\hat{C}_m=\sqrt{\Gamma _m} \hat{\sigma }$ is jump operator regarding of dissipative channels. Then wavefunction obeys Schrödinger equation

6 $$\begin{aligned} \frac{\partial }{\partial t}|\psi (t) \rangle = -i \hat{H}_e |\psi (t) \rangle . \end{aligned}$$

One can obtain $|\psi (t + \delta t) \rangle$ under first-order approximation for sufficiently small $\delta t$

7 $$\begin{aligned} |\psi ^{(1)}(t+\delta t) \rangle = (1 - i \hat{H}_e \delta t)|\psi (t) \rangle . \end{aligned}$$

Notice that Eq. (7) is non-conserved and its norm shows $\Vert |\psi ^{(1)}(t+\delta t) \rangle \Vert \approx 1-\delta p$, where jump probability $\delta p$ defines as

8 $$\begin{aligned} \begin{aligned} \delta p&= \delta t \frac{i}{\hbar }\langle \psi (t)|\hat{H}_e - \hat{H}_e^\dagger |\psi (t) \rangle \\&= \sum _m \delta t \langle \psi (t)| \hat{C}_m^\dagger \hat{C}_m |\psi (t) \rangle \equiv \sum _m \delta p_m, \end{aligned} \end{aligned}$$

which sums over all jump probabilities via channels *m* with $\delta p_m \ge 0$. According to MCWF procedure ^48–51^, quantum jump occurs randomly in each time step with probability $\delta p$. The following evolution is determined by two steps. If no jump occurs, the wavefunction at $t+\delta t$ is renormalized smoothly

9 $$\begin{aligned} |\psi (t+\delta t) \rangle _{\text {n}} = \frac{|\psi ^{(1)}(t+\delta t) \rangle }{\sqrt{1-\delta p}}. \end{aligned}$$

Otherwise, a quantum jump of one specific channel *n* occurs with probability $$\delta 
p_n$$. The wavefunction is interrupted by discontinuous renormalization of population

10 $$\begin{aligned} |\psi (t+\delta t) \rangle _{\text {j}} = \frac{\hat{C}_n |\psi (t) \rangle }{\sqrt{\delta p_n/\delta t}}, \end{aligned}$$

which collapses to final state $\hat{C}_n |\psi (t) \rangle$. In our case of norm-nonconserving MCWF, final states via Auger decay and further photoionization are excluded in reduced Hilbert space. Correspondingly, $|\psi (t') \rangle _{\text {j}} = 0~(t'>t)$ is determined after this jump, which represents complete population leak. And the following evolution of Eq. (6) can be excluded. We emphasize the large efficiency improvement can be accomplished in our simulations, which shows excellent capacity to other complex molecules.

By averaging paths of ensemble at $t+\delta t$, MCWF and QME are indeed equivalent descriptions under Born-Markov approximation ^49^. It concludes that physical property *A*(*t*) is obtained by averaged results of multiple trajectories

11 $$\begin{aligned} A_{n} (t)=\frac{1}{n}\sum _{i=1}^{n}\langle \psi ^{(i)} (t) |\hat{A}| \psi ^{(i)} (t)\rangle . \end{aligned}$$

Here index *i* labels the individual trajectory. *n* is sampling number and $A_n(t) \approx A(t)$ for sufficiently large *n*. Correspondingly, one can obtain standard deviation of *n*-th sample

12 $$\begin{aligned} \delta A_{n} (t)=\Delta A_{n} (t)/\sqrt{n}, \end{aligned}$$

where $\Delta A_{n} (t)$ represents the square root of sample variance

13 $$\begin{aligned} \left[ \Delta A_{(n)} (t)\right] ^2=\frac{1}{n} \sum _{i=1}^{n} \langle \psi ^{(i)} (t) |\hat{A}| \psi ^{(i)} (t)\rangle ^2 - A^2_{n}(t). \end{aligned}$$

In simulations, sampling number *n* satisfies the relation $\sqrt{n} \gg \Delta A_{n} (t) / A (t)$ for high signal-to-noise ratio. Also, *n* should not be too large for computational efficiency. Value of *n* specifically depends on relevant dynamical processes and calculated properties ^49^.

### Transient response function

As for spectral response function, one should obtain expectation value of dipole moment *d*(*t*) from Eq. (11). According to Beer-Lambert formula, spectral response of quantum system irradiated by pulses is described by ^56,64^

14 $$\begin{aligned} S(\omega ,\Delta \tau )=2\text {Im}[d(\omega ,\Delta \tau )\mathscr {E}^*(\omega ,\Delta \tau )] . \end{aligned}$$

Here $\mathscr {E}(\omega ,\Delta \tau )$ and $d(\omega ,\Delta \tau )$ are spectral components of input pulses $\mathscr {E}(t)$ and induced dipole *d*(*t*) with $\Delta \tau$, respectively. When $S(\omega ,\Delta \tau ) >0$, it relates to the absorption of pulses and energy flows to electron by resonant excitation. Reversely, $S(\omega ,\Delta \tau ) <0$ means the amplification of pulses and energy flows back to pulses.

In the framework of single-particle responses, emission spectrum of highly excited DCHs after pulse duration is described by spectral dipole moment $\left| d(\omega ,\Delta \tau )\right| =\left| \mathscr {F}[d(t,\Delta \tau )]\right|$ ^45^, where $\mathscr {F}$ represents the Fourier transformation. Notice that relaxation effects are incorporated into time-dependent dipole responses associated with exponential decay. By varying $\Delta \tau$, spectral reshaping, broadening and beating in both absorption and emission spectrum record ultrafast coherent transitions and unravel hidden transient effects in DCHs generation.

### Calculation details

The sketch of Ne’s DCHs generation in AXPPS is depicted in Fig. 1. Notice that pump pulse contributes to main photoionization from [Ne] to SCH $1s^1$. Furthermore, if $\Delta \tau$ is shorter than relaxation time of SCH and probe pulse matches to Rydberg series DCHs transitions $1s^1 \leftrightarrow 1s^0\text {n}p$, population transfer is established in as. In addition, extra two-photon Raman processes, $1s^1 \leftrightarrow 2p^5 \leftrightarrow 1s^12p^5 \text {n}p$ and $1s^1 \leftrightarrow 1s^0 \text {n}p \leftrightarrow 1s^12p^5 \text {n}p$, are incorporated with additional ultrafast phase modulations.

In simulations, parameters of energy levels, electric dipole moments and transition rates are calculated by flexible atomic code (FAC) with relativistic effects and configuration interactions ^78^, shown in SI.A. 84 states with Rydberg series up to $\text {n}=6$ are included in basis set, in the fact that gap higher than $1s^06p$ is close to natural width and resonant lines become blurred. For the simulation of SASE mode in most XFEL facilities, partial-coherence method is introduced to generate shot-to-shot temporal fluctuations with average spike $\tau _s = 0.26$ fs ^54^.

**Figure 11:**
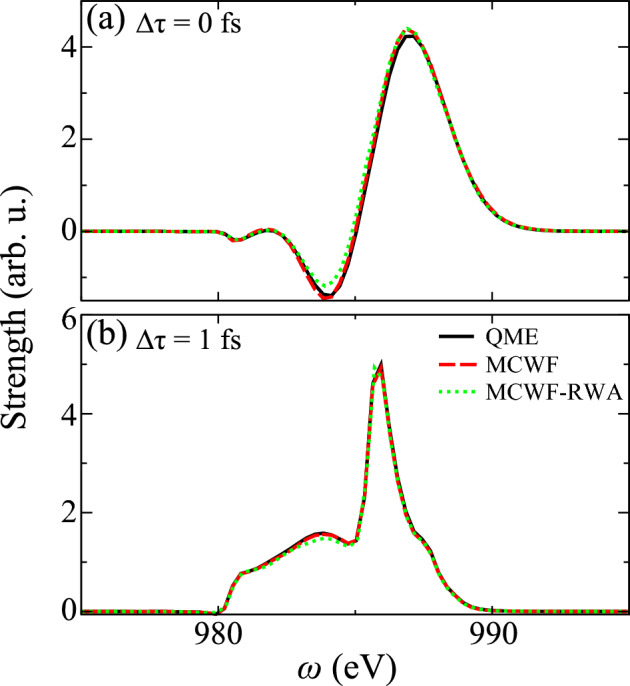
Absorption function $S(\omega ,\Delta \tau )$ of DCHs generation at fixed $\Delta \tau$, (**a**) $\Delta \tau =0$ fs and (**b**) $\Delta \tau =1$ fs. Results of three methods are illustrated, including QME (black solid line), MCWF (red dashed line) and MCWF with RWA (green dotted line). Notice that 2500 samples are averaged in MCWF and only value of MCWF-RWA is scaled to the peak value of MCWF.

### Computational methods test

For fidelity of Monte-Carlo sampling and role of rotating-wave approximation (RWA), absorption functions $S(\omega ,\Delta \tau )$ of QME, MCWF and MCWF-RWA are illustrated in Fig. 11. Simulations are implemented by two Gaussian-profile lasers of pump pulse with photon energy $\omega _1=935$ eV, intensity $I_1=10^{18}$ W/cm$^2$, duration $\tau _1=1$ fs and probe pulse with $\omega _2=986$ eV, $I_2=10^{17}$ W/cm$^2$ and $\tau _2=0.5$ fs, respectively. Notice that parameters of two x-ray lasers are in the range of current double-pulse-generation experiments ^33^. In Fig. 11b, positive delay represents pump laser arrives before probe laser, which contributes to strong absorption in transient responses. Positive peak in 986 eV shows resonance effect. And plateau between 980 and 990 eV is determined by broadening and shifts. Figure 11a illustrates complex transient absorption/emission profile during the overlap, and resonant peak shifts up to 988 eV.

Since MCWF samples decay channels with random jump events, which shows the rough curve in exponential decay region of time domain. According to Fourier relation, fluctuation in exponential tail influences peak width in frequency range. Nevertheless, MCWF results in Fig. 11 clearly show almost identical curves in zero and positive delay compared with QME, even without scaling procedure. Combined with error bar $<1\%$ of population in Fig. 2, we can conclude that 2500 samples are adequate to present reliable and convergent results, due to that coherent dynamics dominates over incoherence of quantum jump. As for time-consuming tests, QME runs about 10 h per delay point under our computational sources, while norm-nonconserving MCWF method needs only half hour with dozens of efficiency increment. Due to superiority of MCWF method in efficiency and reliability, we perform this method in this work and intend to simulate large-scale dynamics in molecules, strongly determined by non-Hermitian dynamics with multi-channel effects, many-body correlations and decoherence.

As for the role of RWA, simulations of “MCWF-RWA” discard fast oscillations of interactions induced by probe laser. Results in Fig. 11 show same curves compared with QME and MCWF at first glance. It derives from large energy gap between SCHs and DCHs, which determines small ratio $\Omega / \omega$ and contribution of counter-rotating term is limited ^79^. However, one can notice that wing and off-resonant peak in spectrum of 983–985 eV have some deviations due to plenty of Raman channels and complex couplings in our model. It illustrates accuracy of RWA is confined within near resonant region, and difference has been observed in our tests of off-resonant condition with strong intensity, e.g. four-wave mixing cases. In the following simulations, results are performed by 2500-averaged MCWF *without* RWA, since computational efficiency and accuracy can be guaranteed. Time step $dt=0.026$ as should be smaller than inverse of eigenvalue energies.

### Supplementary Information

Supplementary Information.

## Data Availability

The datasets generated during and/or analyzed during the current study are available from the corresponding author on reasonable request.
