# Supplementary material for: Transient responses of double core-holes generation in all-attosecond pump-probe spectroscopy
Source: Sci Rep. 2024 Jan 23;14:1950. doi: 10.1038/s41598-024-52197-y (PMC11226462; doi:10.1038/s41598-024-52197-y)
Supplement: Supplementary file 1 — Supplementary Information. [file 41598_2024_52197_MOESM1_ESM.pdf]

Supplementary Information

Atomic parameters

To present atomic data in our simulations, Table S1 and S2 contain parameters about bound-bound and bound-free transitions, respectively. Three types of transitions, illustrated in Fig. 1 of main text with purple hollow arrows, are listed in Table S1. Due to diverse final states of Raman channels $1s^1 2p^5 \mathrm{n}p$, here we present averaged value of all configurations for clarification. As for decay channels, Table S2 presents two types of decoherent channels. One can see pump rates of K-shell photoionization to SCH $1s^1\ ^2\mathrm{S}_{1/2}$ dominate pump processes. Main dissipation derives from further photoionization and Auger decay, with 2 orders of magnitude larger than spontaneous decay at least. And both of them show significant influence on evolution. For clarity without loss of generality, we include all of them in simulations.

Table S1. Oscillation strength gf, spontaneous decay γ (Hz) and transition energy ΔE (eV) of bound-bound channels are listed. Three types of transitions, illustrated in Fig. 1 of main text with purple hollow arrows, are shown separately. Value in square brackets represents multiplication by powers of 10. For clarification, averaged value of configurations $1s^1 2p^5 \mathrm{n}p$ is presented.

Transitions		gf	γ	ΔE
**I. SCHs $\leftrightarrow$ DCHs**				
$1s^1\ ^2\mathrm{S}_{1/2}$	$\leftrightarrow 1s^0 3p\ ^2\mathrm{P}^{\mathrm{o}}_{1/2}$	9.06[-3]	1.89[11]	980.44
	$\leftrightarrow 1s^0 3p\ ^2\mathrm{P}^{\mathrm{o}}_{3/2}$	1.81[-2]	1.89[11]	980.44
$1s^1\ ^2\mathrm{S}_{1/2}$	$\leftrightarrow 1s^0 4p\ ^2\mathrm{P}^{\mathrm{o}}_{1/2}$	1.76[-3]	3.71[10]	985.64
	$\leftrightarrow 1s^0 4p\ ^2\mathrm{P}^{\mathrm{o}}_{3/2}$	3.51[-3]	3.70[10]	985.64
$1s^1\ ^2\mathrm{S}_{1/2}$	$\leftrightarrow 1s^0 5p\ ^2\mathrm{P}^{\mathrm{o}}_{1/2}$	6.43[-4]	1.36[10]	987.64
	$\leftrightarrow 1s^0 5p\ ^2\mathrm{P}^{\mathrm{o}}_{3/2}$	1.28[-3]	1.36[10]	987.64
$1s^1\ ^2\mathrm{S}_{1/2}$	$\leftrightarrow 1s^0 6p\ ^2\mathrm{P}^{\mathrm{o}}_{1/2}$	2.64[-4]	5.61[9]	988.64
	$\leftrightarrow 1s^0 6p\ ^2\mathrm{P}^{\mathrm{o}}_{3/2}$	5.28[-4]	5.60[9]	988.64
**II. Raman channels** $1s^1 \leftrightarrow 2p^5 \leftrightarrow 1s^1 2p^5 \mathrm{n}p$				
$1s^1\ ^2\mathrm{S}_{1/2}$	$\leftrightarrow 2p^5\ ^2\mathrm{P}^{\mathrm{o}}_{3/2}$	2.50[-1]	3.93[12]	851.18
	$\leftrightarrow 2p^5\ ^2\mathrm{P}^{\mathrm{o}}_{1/2}$	1.25[-1]	1.97[12]	851.09
$2p^5\ ^2\mathrm{P}^{\mathrm{o}}_{3/2}$	$\leftrightarrow 1s^1 2p^5 \mathrm{n}p$	2.64[-3]	2.27[10]	889.89
$2p^5\ ^2\mathrm{P}^{\mathrm{o}}_{1/2}$	$\leftrightarrow 1s^1 2p^5 \mathrm{n}p$	1.76[-3]	1.98[10]	890.45
**III. Raman channels** $1s^1 \leftrightarrow 1s^0 \mathrm{n}p \leftrightarrow 1s^1 2p^5 \mathrm{n}p$				
$1s^0 3p$	$\leftrightarrow 1s^1 2p^5 \mathrm{n}p$	4.49[-2]	5.78[11]	940.06
$1s^0 4p$	$\leftrightarrow 1s^1 2p^5 \mathrm{n}p$	5.02[-2]	6.46[11]	944.37
$1s^0 5p$	$\leftrightarrow 1s^1 2p^5 \mathrm{n}p$	6.12[-2]	7.88[11]	945.71
$1s^0 6p$	$\leftrightarrow 1s^1 2p^5 \mathrm{n}p$	7.60[-2]	9.79[11]	946.08

Proof of positive Stark shifts

In this subsection, we present qualitative proof of positive Stark shifts in two Raman channels $2p^5 \leftrightarrow 1s^1 \leftrightarrow 1s^0 \mathrm{n}p$ and $1s^1 \leftrightarrow 1s^0 \mathrm{n}p \leftrightarrow 1s^1 2p^5 \mathrm{n}p$. Fig. S1(a) shows the simplified cascading three-level model with respect to former Raman channel in degenerate condition. Here $|0\rangle$, $|1\rangle$ and $|2\rangle$ respectively label $2p^5$, $1s^1$ and $1s^0 \mathrm{n}p$. Resonant conditions of two lasers satisfy $\omega_0 = \Delta E_{10} + \Delta$ and $\omega_1 = \Delta E_{12} + \delta - \Delta$. The Hamiltonian of atom-laser interactions describes as

$$\hat{H}'(t) = -\frac{1}{2}\left[\Omega_0 e^{-i\phi_0(t)}|1\rangle\langle 0| + \Omega_1 e^{-i\phi_1(t)}|2\rangle\langle 1|\right] + \mathrm{H.c.} \tag{S1}$$

Table S2. Maximum transition rates by pump laser Γ_1 (Hz) with $I_1 = 10^{18}$ W/cm^2 and by probe laser Γ_2 (Hz) with $I_2 = 10^{17}$ W/cm^2, Auger decay Γ_A (Hz) and further total photonionization rates are listed, corresponding to blue, green and black arrows in Fig. 1 of main text. Value in square brackets represents multiplication by powers of 10. For clarification, averaged value of configurations $1s^1 2p^5 \mathrm{n}p$ is presented and $J = 1/2,\ 3/2$.

Transitions		Γ_1 (Hz)	Γ_2 (Hz)	Γ_A (Hz)
**I. Pump channels**				
[Ne] $^1\mathrm{S}_0$	$\rightarrow 2p^5\ ^2\mathrm{P}^o_{3/2}$	4.03[13]	3.23[12]	-
	$\rightarrow 2p^5\ ^2\mathrm{P}^o_{1/2}$	2.04[13]	1.64[12]	-
	$\rightarrow 1s^1\ ^2\mathrm{S}_{1/2}$	1.59[15]	1.32[14]	-
	$\rightarrow 1s^1 2p^5 \mathrm{n}p$	4.19[11]	1.75[11]	-
**II. Dissipative channels**				
[Ne] $^1\mathrm{S}_0$	$\rightarrow$ others	8.80[13]	7.40[12]	-
$2p^5\ ^2\mathrm{P}^o_J$	$\rightarrow$ others	1.36[15]	1.21[14]	-
$1s^1\ ^2\mathrm{S}_{1/2}$	$\rightarrow$ others	1.70[14]	1.40[13]	3.79[14]
$1s^0 3p\ ^2\mathrm{P}^o_J$	$\rightarrow$ others	1.90[14]	1.56[13]	9.74[14]
$1s^0 4p\ ^2\mathrm{P}^o_J$	$\rightarrow$ others	1.93[14]	1.58[13]	9.71[14]
$1s^0 5p\ ^2\mathrm{P}^o_J$	$\rightarrow$ others	1.94[14]	1.59[13]	9.70[14]
$1s^0 6p\ ^2\mathrm{P}^o_J$	$\rightarrow$ others	1.92[14]	1.57[13]	9.82[14]
$1s^1 2p^5 \mathrm{n}p$	$\rightarrow$ others	1.74[14]	1.44[13]	3.57[14]

with phases $\phi_i(t) = \omega_i t + \phi_i$ $(i = 1,2)$. Considering wavefunction is expanded as $|\psi(t)\rangle = c_0(t)|0\rangle + c_1(t)|1\rangle + c_2(t)|2\rangle$, variable replacements of $a_i(t) = c_i(t)e^{iE_i t}$ $(i = 0,2)$ and $a_1(t) = c_1(t)e^{i(E_1+\Delta)t}$ are introduced and final equations are

$$\begin{cases} \dot{a}_0(t) = \dfrac{i}{2}\Omega_0 e^{i\phi_0} a_1(t) \\ \dot{a}_1(t) - i\Delta a_1(t) = \dfrac{i}{2}\left[\Omega_0 e^{-i\phi_0} a_0(t) + \Omega_1 e^{i(\delta t+\phi_1)} a_2(t)\right] \\ \dot{a}_2(t) = \dfrac{i}{2}\Omega_1 e^{-i(\delta t+\phi_1)} a_1(t). \end{cases} \tag{S2}$$

Since coupling $|0\rangle \leftrightarrow |1\rangle$ is mainly induced by pump laser in our case, $\Delta \approx 84$ eV is much larger than Auger decay rates of SCHs and Rabi frequency $\Omega_{0,1}$ and $|\dot{a}_1(t)| \ll |\Delta a_1(t)|$, which evolution of $a_1(t)$ is adiabatically followed by $a_0(t)$ and $a_2(t)$. Hence

$$a_1(t) \approx -\frac{1}{2\Delta}\left[\Omega_0 e^{-i\phi_0} a_0(t) + \Omega_1 e^{i(\delta t+\phi_1)} a_2(t)\right] \tag{S3}$$

and further bring back into Eq. (S2). One can obtain

$$\begin{cases} \dot{a}_0(t) = -\dfrac{i}{4\Delta}\left[\Omega_0^2 a_0(t) + \Omega_0\Omega_1 e^{i(\delta t+\phi)} a_2(t)\right] \\ \dot{a}_2(t) = -\dfrac{i}{4\Delta}\left[\Omega_0\Omega_1 e^{-i(\delta t+\phi)} a_0(t) + \Omega_1^2 a_2(t)\right], \end{cases} \tag{S4}$$

where $\phi = \phi_1 + \phi_2$. We define two-photon Rabi frequency $\Omega = \Omega_0\Omega_1/2\Delta$ and Stark shift $\delta_i = \Omega_i^2/4\Delta$ $(i = 0,1)$. One can see Stark shifts of two states in Eq. (S4) have same sign by additional coupling $|0\rangle \leftrightarrow |1\rangle$. And when $\Delta > 0$, it introduces positive shift $\delta_2 = \Omega_2^2/4\Delta > 0$ to $1s^0 \mathrm{n}p$, and transition energy of $1s^1\ ^2\mathrm{S}_{1/2} \leftrightarrow 1s^0 \mathrm{n}p\ ^2\mathrm{P}^o$ adiabatically shifts to higher energy at around zero delay in Fig. 4(c) of main text.

Similar deduction is made in Λ-type three-level model, which $|0\rangle$, $|1\rangle$ and $|2\rangle$ respectively label $1s^1 2p^5 \mathrm{n}p$, $1s^0 \mathrm{n}p$ and $1s^1$ in Fig. S1(b). DCHs $1s^0 \mathrm{n}p$ are effectively eliminated, by coupling $|0\rangle \leftrightarrow |1\rangle$ with detuning $\Delta \approx -10$ eV. On the basis of deduction as above, SCH $1s^1$ has negative shifts $\delta_2 < 0$ and transition of DCHs shifts to higher energy, which is consistent with results in Fig. 4(b) of main text. Despite complicated multi-channel Λ-type model and effective cross coupling among diverse transitions

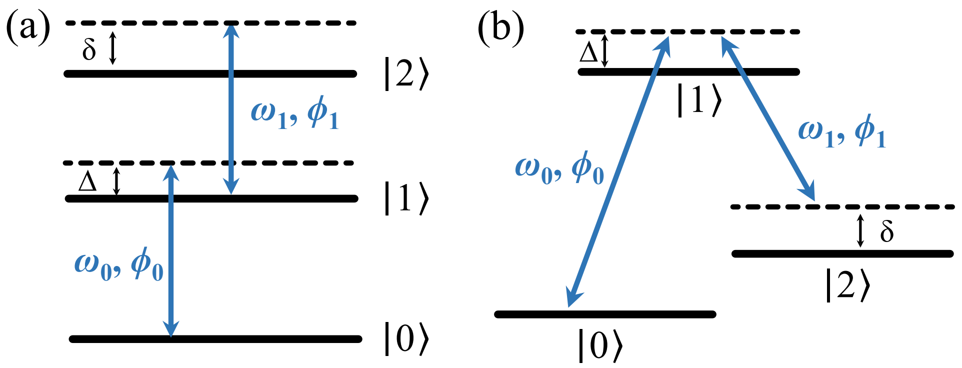

Figure S1. (a) and (b) show diagram of cascading three-level model and Λ-type three-level model, respectively. Here $|0\rangle$, $|1\rangle$ and $|2\rangle$ label three states. $\omega_{0,1}$ and $\phi_{0,1}$ label photon energy and phase of two pulses, of which subscripts $\{0,1\}$ represent transitions $|0\rangle \leftrightarrow |1\rangle$ and $|1\rangle \leftrightarrow |2\rangle$ with detuning Δ and δ, respectively.

exclude explicit resolution, each individual Λ-type three-level models of $1s^0\mathrm{n}p \leftrightarrow 1s^1 2p^5 \mathrm{n}p$ obey $\Delta < 0$ and negative shifts of all transitions remain when neglecting higher-order Stark shifts.
